# The ongoing challenge of latent tuberculosis

**DOI:** 10.1098/rstb.2013.0437

**Published:** 2014-06-19

**Authors:** H. Esmail, C. E. Barry, D. B. Young, R. J. Wilkinson

**Affiliations:** 1 Department of Medicine, Imperial College, London W2 1PG, UK; 2 Clinical Infectious Diseases Research Initiative, Institute of Infectious Diseases and Molecular Medicine, University of Cape Town, Observatory 7925, South Africa; 3 Tuberculosis Research Section, NIAID, NIH, Bethesda, MD 20892, USA; 4 MRC National Institute for Medical Research, London NW7 1AA, UK

**Keywords:** latent tuberculosis, *Mycobacterium tuberculosis*, elimination, diagnosis, treatment, natural history

## Abstract

The global health community has set itself the task of eliminating tuberculosis (TB) as a public health problem by 2050. Although progress has been made in global TB control, the current decline in incidence of 2% yr^−1^ is far from the rate needed to achieve this. If we are to succeed in this endeavour, new strategies to reduce the reservoir of latently infected persons (from which new cases arise) would be advantageous. However, ascertainment of the extent and risk posed by this group is poor. The current diagnostics tests (tuberculin skin test and interferon-gamma release assays) poorly predict who will develop active disease and the therapeutic options available are not optimal for the scale of the intervention that may be required. In this article, we outline a basis for our current understanding of latent TB and highlight areas where innovation leading to development of novel diagnostic tests, drug regimens and vaccines may assist progress. We argue that the pool of individuals at high risk of progression may be significantly smaller than the 2.33 billion thought to be immune sensitized by *Mycobacterium tuberculosis* and that identifying and targeting this group will be an important strategy in the road to elimination.

## 1. Introduction

*Mycobacterium tuberculosis* (Mtb) is a pathogen that has coevolved with anatomically modern humans [1–3], co-migrating from Africa as our population expanded to cover every area of the globe (see box 1). It has been estimated that in 2006 there were more cases of tuberculosis (TB) than in any other year in recent history [7], and yet the ambitious vision adopted by the World Health Organization (WHO) and Stop TB partnership is to eliminate TB as a public health problem by 2050 [8]. This has been defined as achieving an incidence rate of less than 1 case per million of the global population; for comparison, the 2012 rate is 1220 cases per million [9]. Various regions of the world are in different phases of the TB epidemic and will require different strategies to make progress towards elimination. In the twentieth century, much of Western Europe, North America and parts of East Asia saw dramatic reductions in TB incidence through major social and economic progress and implementation of improved TB control and treatment programmes, with reduction in TB cases of up to 8.8% yr^−1^ being achieved after the second world war [10]. By contrast, sub-Saharan Africa and Eastern Europe/Central Asia in particular suffered a steep increase in incidence during the 1990s owing to the HIV epidemic and the social and economic disruption following collapse of the Soviet Union, respectively.

Box 1. **Origins and evolution of latency in tuberculosis.**

Current evidence suggests that Mtb was already established as an infection of ancient human populations prior to migration out of Africa. In these small isolated hunter–gatherer populations, sustained infection would be favoured by low-virulence pathogens capable of persisting within the human host by chronic or latent infection and transmitting to susceptible new birth cohorts years or decades after initial infection. Higher virulence pathogens with shorter incubation would result in self-terminating epidemics owing to elimination of susceptible hosts [2,4]. It has been speculated that increases in human population density associated with the Neolithic Revolution in farming and the Industrial Revolution in Europe may have favoured the emergence of Mtb strains with greater virulence and shorter incubation periods [1,3,5]. According to this model, carriage as an asymptomatic commensal may have been the predominant mode of Mtb infection in ancient human populations and may have shaped the natural immune response. The current predominant high mortality form of TB would then represent a relatively recent challenge to human health. This model is consistent with phylogenetic analysis of global Mtb and with epidemiological differences between the spread of ‘modern’ Beijing strains and that of ‘ancient’ *Mycobacterium africanum* [6].

### (a) Targeting tuberculosis

Progress recently has certainly been made in global TB control aided by a number of internationally agreed targets over the last two decades. In the 1990s, as part of the WHO DOTS strategy, a commitment to identify 70% and cure 85% of TB cases by 2005 was made, and this was largely achieved in many parts of the world [11]. Subsequently, as part of the response to the United Nations Millennium Development Goal 6 to combat HIV/AIDS, malaria and other major diseases, the Stop TB partnership set a target of reducing the global mortality and prevalence of TB disease by 50% compared with 1990 levels. By 2012, a 37% reduction in global prevalence of TB had been achieved (although not on track to achieve 50% reduction by 2015) and a 45% reduction in mortality (on track to achieve 50% reduction by 2015) [12]. Latest estimates suggest that in 2012 there were 8.6 million new cases of TB and 1.3 million deaths, with the global incidence of TB falling 2% yr^−1^ over recent years [9]. In this context, the 2050 elimination target seems particularly bold, requiring a historically unprecedented 20% yr^−1^ reduction in global incidence [10]. A detailed strategy to make the 2050 vision a reality is currently being developed and will be announced in 2014 with interim targets for 2025 and 2035 being proposed. In the initial phase, scale-up and widespread implementation of current TB control measures coupled with continued socioeconomic development particularly within the BRICS (Brazil, Russia, India, China and South Africa) countries along with continued antiretroviral therapy (ART) roll out in sub-Saharan Africa could result in reductions in TB incidence of 10% yr^−1^. However, to bring global incidence down towards current levels seen in North America and parts of Western Europe, considered to be in the elimination phase (less than 100 cases/million yr^−1^), by 2035 will require development of novel technologies and approaches though research and innovation. Whereas until now TB control has focused on detection and management of active disease, which will continue to be important, a renewed focus on understanding and managing the important reservoir of infected humans with latent infection will be critical to future progress. Modelling suggests that mass treatment of latent TB would be one of the most effective ways to reduce incidence of TB [10,13], but with current treatment and diagnostics this would involve up to one-third of the world's population taking three to nine months of anti-tuberculous therapy, which is neither desirable or feasible. In this article, we will highlight our current understanding of latent TB and the gaps in our knowledge that need to be filled to develop more predictive diagnostic tests, effective short-course treatments and vaccines.

## 2. Diagnosing latent infection

Latent tuberculosis may be defined for convenience as that which is unaccompanied by symptoms and physical signs, causes no obvious disturbance and is not recognised by the physician. There is no sharp distinction between latent and manifest tuberculosis, and in some instances latent tuberculosis is more extensive than that which is recognisable. Ability to distinguish between latent and manifest disease will vary with the means available for diagnosis. (Opie & McPhedran 1926 [14, p. 347])

Opie & McPhedran's [14] characterization of latent TB remains accurate and insightful 88 years after writing, and it is striking that the means physicians have available to distinguish active and latent TB remain essentially unchanged today. Chest X-ray, tuberculin skin testing and sputum investigation for evidence of Mtb remain the cornerstones of diagnosis. Interferon-gamma release assays developed over the last decade represent an evolution in diagnosis of latent TB rather than a conceptual shift as they also indicate immune sensitization. Advanced imaging such as computed tomography (CT) and combined positron emission tomography/computed tomography (PET/CT) used to identify minimal disease and invasive sampling also now play a role in specialist centres in high-income countries in selected cases. The diagnostic pathway nevertheless still ends in a binary classification with those considered latently infected being potentially eligible for preventive therapy and those with active disease, standardized treatment.

### (a) The development of the tuberculin skin test

By the end of the nineteenth century, the notion that a latent period of infection could occur in TB was widely accepted. Several autopsy case series carried out around the turn of the century demonstrated that Mtb was frequently present in persons who died of causes other than TB by inoculating material into rabbits or guinea pigs, where secondary infection was observed [15,16]. Detecting the presence of infection in asymptomatic living people was more challenging. Koch's discovery and development of tuberculin, a heat-killed culture filtrate of TB that was proposed unsuccessfully as a cure in 1890, provided a useful diagnostic test, unmasking occult infection by inducing systemic reaction following subcutaneous injection [17]. Over a period of 60 years, this was refined into an intradermal skin test using a standardized purified protein derivative of tuberculin (PPD) and measuring the induration formed after 48–72 h. The result, expressed as millimetres (mm) of induration, is a continuous variable where the threshold for a positive result can be varied to modify diagnostic sensitivity and specificity. The dose of PPD was optimized to maximize sensitivity in distinguishing healthy close contacts of TB from healthy non-contacts, and the tuberculin skin test (TST) remains widely used globally today and is generally considered to demonstrate the presence of infection [18].

But to what extent can this relationship be assumed to be true in the obvious absence of autopsy studies to relate premorbid TST to post-mortem identification of viable bacilli? The guinea pig ‘natural infection’ model, initially developed by Riley to investigated airborne transmission in which air from side rooms or wards where TB-infected patients are resident is vented over chambers housing guinea pigs, can provide some evidence. In these studies, the distribution and magnitude of TST reactions found in guinea pigs was similar to humans; at autopsy, evidence of infection was found in 0% of guinea pigs with tuberculin reactions of 0–5 mm (negative reaction), 92% of guinea pigs with TST of 14 mm or more or evidence of necrosis, but only 25% with TST of 6–13 mm [19]. This finding, that not all ‘naturally infected’ guinea pigs with positive TST have evidence of infection at autopsy, was subsequently confirmed by others [20,21]. Equally, if positive TST indicated presence of infection and negative TST absence of infection, then treatment of latent infection might be expected to cause a reversion of status. In the United States Public Health Service Trials 13 176 household contacts who were initially tuberculin reactors had TST repeated at 12 months; 6.5% of contacts who received placebo converted to negative and 7.9% of isoniazid treated subjects converted to negative. However, isoniazid reduced the 10-year incidence of TB by 59% in those that remained TST positive at 12 months and by 38% in those that converted at 12 months, indicating that isoniazid's efficacy to prevent disease was not associated with a capacity to induce TST reversion [22]. From this, we conclude that TST provides evidence of immune sensitization by Mtb and is a correlate of TB infection but usually remains positive even if infection is treated.

Because TST can register low-level false positive results due to sensitization by environmental bacteria and BCG, interferon (IFN)-gamma release assays (IGRAs) were developed to improve specificity of the diagnosis. While these tests provide a reasonably good measure of TB exposure and their negative predictive values are very high (IGRA 99.7%, TST 99.4%), they are, like TST, poorly predictive of progression to active disease (positive predictive value: IGRA 2.7%, TST 1.5%) [23,24].

As a result, when these immunodiagnostic tests are used as a guide for administration of preventive therapy, the number needed to treat (NNT) to prevent one case of active TB is high. In a systematic review of 11 studies involving 73 375 HIV-uninfected participants where presence of infection was mainly determined by TST and participants were randomized to isoniazid or placebo, the pooled NNT was 100, ranging from 36 in recently infected household contacts and up to 179 in those remotely infected [25]. After the initial need to develop sensitive and then specific tests for latent TB, we now need to develop tests that are better able to predict who will develop active TB.

## 3. Estimating the global burden of latent tuberculosis

Defining the prevalence of infection at a global and a regional level is critical to understanding the potential size of the reservoir of infection and planning intervention strategies. One of the most widely quoted statistics is that one-third of the world's population is infected by Mtb, emphasizing the huge scale of the problem [26]. However, no test actually demonstrates the presence of infection and it is useful to consider the data upon which the statement is made. Prevalence of infection in a population is not directly estimated from population-wide tuberculin surveys but derived from the annual rate of infection (ARI), which can be either directly determined from focused tuberculin surveys (usually in school children) or indirectly estimated from incidence of active TB (itself usually estimated from case notification, disease prevalence or mortality data), using the equation ARI = incidence/coefficient, as risk of infection is determined by contact with infectious cases [27]. In addition, the change in ARI over time should be known in order to accurately determine prevalence of infection throughout the population.

In 1999, WHO convened a consensus group comprising 86 experts and epidemiologists who evaluated the best available data for all countries up to 1997 for a number of TB indicators including prevalence of infection [26]. Recent good quality tuberculin surveys were available for only 24 countries and the rate of change in ARI was only accurately known in a minority. For the majority of countries for which good quality tuberculin surveys were not available (or it was not possible to confidently extrapolate from countries with good data), ARI was derived from the incidence of smear positive disease using the equation above with the coefficient of 50 (for countries where HIV prevalence in TB cases was less than 5%) coming from ‘Styblo's rule’ [28] (which states that a smear positive pulmonary TB incidence of 50/100 000 yr^−1^ corresponds to ARI of 1%, potentially an overestimate—*vide infra*). The authors estimated that 32% of the world's population was infected with Mtb but acknowledged the lack of good data and limitations of the models to determine prevalence of infection and did not provide an uncertainty estimate.

Tuberculin surveys, while widely performed and a potentially cost effective way to monitor changes in the burden of infection, have several limitations. Aside from the inherent issue of observer variability, interpreting results to determine the proportion that are immune sensitized by Mtb is challenging as the specificity of the test varies between populations depending upon exposure to environmental mycobacteria and BCG vaccination. Analysing the distribution of TST reactions using mixture models and other techniques to identify bimodal patterns (as the reaction to TST is greater following immune sensitization by Mtb than environmental mycobacteria or BCG) can be performed but not all distributions lend themselves to this form of analysis [29,30]. Systematic surveys using IGRA have not often been performed and the need for venepuncture, specialist laboratories and cost may limit widespread use. In addition, the dynamics of IGRA conversion and reversion over time and hence sensitivity to detect remote infection are less well understood, although data from IGRA surveys could be used with tuberculin surveys to refine estimates of latent TB infection (LTBI) prevalence [31]. A recombinant ESAT-6/CFP-10 skin test is currently in development with early clinical studies showing superior specificity to TST and correlation with whole blood Quantiferon results [32]; such a test may ultimately prove particularly useful for surveys of this kind.

Modelling the risk of infection from the incidence of smear positive active disease seems appropriate but ‘Styblo's rule’ makes some key assumptions informed by six studies between 1921 and 1971: each smear positive incident case is infectious for 2 years (corresponding to two prevalent cases), and each prevalent case results in 10 new infections per year. Hence the rule, an incidence of 50/100 000 yr^−1^ results in an infection rate of 1000/100 000 yr^−1^. This may be less applicable in the contemporary era as improved diagnosis and treatment may have reduced the average duration of infectiousness significantly and factors such as population density, success of TB control programmes, the prevalence of HIV infection and the prevalence of drug-resistant TB will also have influenced transmission [33]. A recent analysis of data from East Asian countries between 1975 and 1994 by van Leth *et al*. [34] determined that the number of infections per prevalent smear positive case was 2.6–5.9 yr^−1^, so at least in some parts of the world ‘Styblo's rule’ overestimates infection [33,34]. Smear positive TB is also not homogeneous. Jones-Lopez *et al*. [35] have shown that in only 45% of smear positive cases could Mtb be cultured from cough aerosols generated through 10 min of strong coughing. In addition, the variability in colony-forming units (cfu) generated was great (1–378 cfu). They also showed that infection of household contacts was significantly greater when the index cases had a high cough aerosol cfu compared with low or no cough aerosol cfu [35]. Confirming that cases of TB transmit variably, Escombe *et al*. [36] using the Riley guinea pig model of airborne infection in an HIV/TB ward in Peru showed that 8.5% of admissions were responsible for 98% of infections in guinea pigs. Further complicating the situation is the fact that although previously Mtb was considered a highly invariant pathogen, recent large-scale whole genome sequencing projects have made it clear that the pathogen has continued to evolve. As human population density has expanded exponentially and living conditions have shifted from low-density agrarian conditions to high-density urban conditions, new genetic variants of Mtb have emerged, displacing formerly resident strains [37] (see box 1). More ‘modern’ strains differ from their ancient progenitors, notably at a cellular level in the magnitude of the innate immune response they elicit [38]. More variation in these strains has been observed than previously expected, and plausible links to enhanced transmissibility have been inferred from cluster size in human populations exposed to multiple strain clades [39]. Comparison of experimental infection outcome in non-human primates with modern and ancient strains reveals strikingly different outcomes of infection among currently prevalent clades [40]. The contribution of strain variability to differences in transmissibility is likely to be geography and population-density specific, and employing any general rule to establish prevalence of infection base on reported cases is likely to be extremely inaccurate.

**Figure 1.**
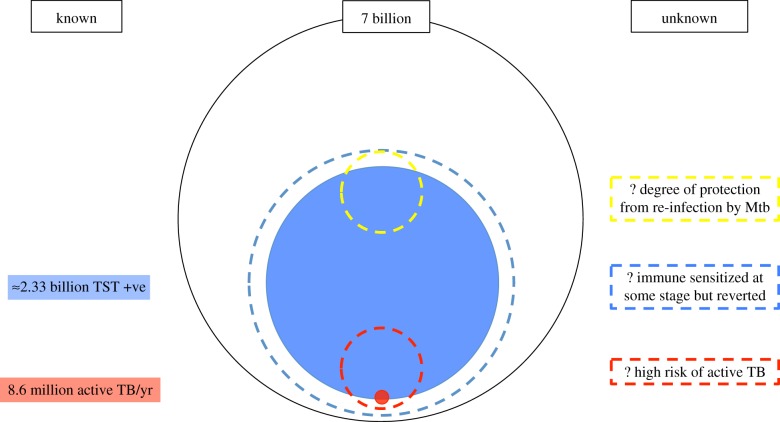
Reservoir of TB—we currently have estimates for proportion of population that are immune sensitized (large circle) and number of cases of active TB annually (small filled circle). As TST and IGRA reversion can occur, total number of exposed persons may be greater than this (larger dashed circle), in addition TST and IGRA are only moderately sensitive for active TB. A much smaller pool of people may be at much higher risk of TB (bottom small dashed circle) and also a proportion of people may receive considerable protection against reinfection (top small dashed circle). Identifying these additional populations may be very valuable. (Online version in colour.)

Accurate estimation of the proportion of the world that is infected using the currently available tools is extremely difficult. Better data and a better understanding of how parameters change regionally or in certain situations (such as drug resistance) may allow for the development of more sophisticated models [41] that will allow more accurate assessment of the prevalence of infection and estimates of useful subgroups such as the prevalence of drug-resistant LTBI or the proportion of LTBI related to a recent infection. However, ultimately what we want to know is the proportion that is highly likely to develop active disease (figure 1).

## 4. The natural history of tuberculosis

The natural history of TB is more complex than most bacterial pathogens. The incubation period is prolonged and the outcome of infection variable depending upon both host and pathogen. Much of our current understanding still arises from piecing together historical studies and evidence from animal models that often fail to replicate key aspects of human disease. However, understanding this natural history of infection is critical to accurate categorization of TB-infected persons, identification of correlates of risk and protection, and development of novel interventions.

Although TB can develop in virtually any part of the body, disease involving the lungs (which occurs in 60–75% of cases) is necessary for transmission of infection, in particular pulmonary cavitation facilitates efficient Mtb replication and transmission. There is some evidence that suggests Mtb may specifically exploit the immune response through conservation of immunodominant epitopes [42], which could promote the induction of immunopathology that leads to lung cavitation. It is immunocompetent adults that contribute most to disease transmission. These individuals who are most effective at transmitting are often sputum smear positive and cough spontaneously, thereby generating infectious particles. Infection is initiated by droplet nuclei of less than 5 µm that can remain suspended in the air for hours (if not disrupted by turbulence) and be inhaled by contacts sharing the same environment [43]. Around 30–50% of close household contacts will develop evidence of immune sensitization as a result of infection [44].

A single droplet nucleus (probably containing 1–10 bacilli) can initiate infection, with the site of implantation following chance distribution strongly influenced by the particle size across the lung lobes [45]. The early stages of infection are characterized by a localized macrophage-rich alveolitis, lymphatic spread to regional mediastinal lymph nodes and a low-grade bacillaemia allowing distant dissemination [46,47]. Approximately 2–10 weeks following initial infection, a cell-mediated immune response develops, signified by tuberculin conversion, facilitating the development of granulomas which promote control of infection [48] (box 2). This initial infection is often asymptomatic but may be associated with fever, mild chest symptoms and increased inflammatory markers [52,53]. The primary infiltrate may be visible on chest radiograph in 2–6% of older children and adults [54] (this may be considerably higher in young children [55,56]). In a small proportion (less than 15%), the visible primary infiltrate may progress (progressive primary TB), but in general the lesion heals and often eventually calcifies. If progressive TB disease subsequently develops within the lungs, it does so at a distant site, most commonly arising apically or sub-apically; the mechanism for this characteristic localization is poorly understood and the source of some speculation [57–59].

### (a) Lifetime risk of infection progressing to disease

**Figure 2.**
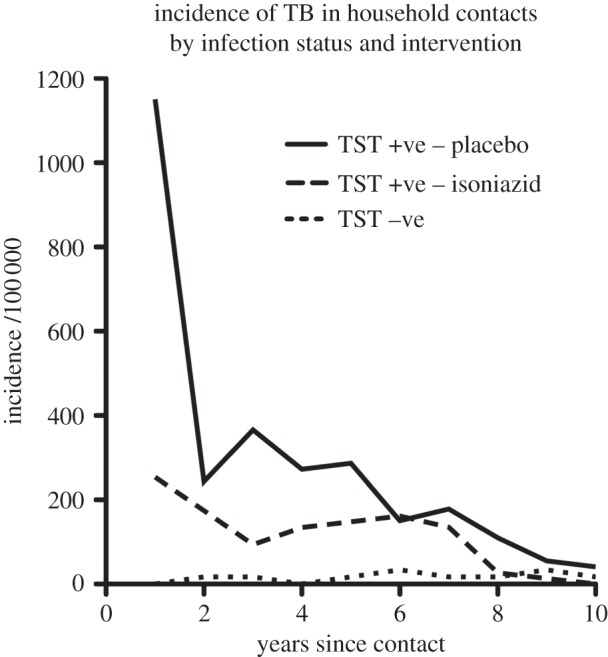
Incidence of TB in household contacts of TB treated with isoniazid or placebo over 10 years by TST status (TST − ve less than 5 mm). Based on data for 26 833 persons from Ferebee [22].

The lifetime age-weighted risk of TB following infection in settings with low exogenous reinfection is estimated to be 12% [60]. Careful follow-up in placebo-controlled intervention studies has demonstrated that disease is most likely to occur in the first year following infection, with stepwise reduction year on year over the following 5–10 years (figure 2), by which time incidence approaches that of uninfected contacts [22]. The different manifestations of TB occur at different intervals following infection, with pleural TB, TB meningitis and miliary TB occurring after a shorter interval than pulmonary or other extra-pulmonary sites.

Reactivation several decades after initial infection occurs [61], but as observational studies with close follow-up rarely continue beyond 10 years it is difficult to assess how common reactivation is outside this timeframe. In addition, conventional observational studies make it difficult to evaluate whether disease relates to the initial infection event or subsequent reinfection. Borgdorff *et al.* [62] applied a molecular epidemiology approach (using restriction fragment length polymorphism of *IS6110* +/− polymorphic GC-rich sequence) to 12 222 cases of TB over a 15-year period in the Netherlands and identified 1095 linked secondary cases from 688 source cases. The median incubation period (time between predicted date of infection and onset of symptoms in the secondary case) was calculated to be 1.26 years, and the serial interval (time between symptom onset in source and secondary case) was found to be 1.44 years with 83% of secondary cases occurring within 5 years of the source case and more than 95% within 10 years [62].

Studies of immigrants from high to low burden countries provide further insight. Risk of TB is especially high in the first few years following migration, but migrants remain at higher risk of TB for decades after entry [63]. It is difficult to establish whether this relates to delayed reactivation or (re)infection following recent transmission either from visiting country of origin or from the local community. However, McCarthy [64] has shown that diagnosing TB in migrants is very rare more than 15 years after arrival if they have low risk of re-exposure. In this study, of 230 migrants from high burden countries in Asia diagnosed with TB in London in the 1980s (low burden setting), 10.4% had arrived in the UK 11–15 years previously and 5.6% more than 15 years previously; however, in those who had never returned to Asia since migrating and had no known UK TB contact, only 3.9% had arrived 11–15 years previously and 0.8% more than 15 years previously [64].

Furthermore, the observation that the elderly in low TB burden settings have a higher incidence of TB is often, possibly incorrectly, interpreted as providing evidence of prolonged latency and reactivation following immunosenescence. However, careful evaluation of birth cohorts shows that this apparent increased risk is an artefact of falling transmission, and younger adults are still invariably at greater risk of TB than the elderly [65]. These data show that the common view that reactivation TB disease often occurs decades after initial infection may be overstated; the majority of cases occur within 18 months of infection and disease resulting from reactivation more than 10 years after infection may be rare. The risk of disease is also not constant over time; following a single infection, the risk is 12% over a person's lifetime; if no disease develops after 5 years the lifetime risk might only be 2% and after 10 years 0.5%.

It is likely that at least in some people for whom there is a prolonged time interval between infection and eventual disease presentation, episodes of subclinical reactivation had occurred much sooner. Evidence for this comes from twentieth century mass chest radiograph (CXR) screening programmes, which identified asymptomatic persons with no previous history of disease but with apical fibrotic scarring felt to represent inactive or arrested TB. These individuals were up to 15 times more likely to develop TB than those having normal CXR, with the risk of developing TB steadily falling over a 5–10 year period of observation [66]. In addition, studies in Europe and America at a time of rapidly falling TB incidence showed that up to 70% of persons developing TB (with no history of TB and usually no clear contact history) had evidence of fibrotic scarring on previous CXR [67,68]. This suggests that, in a proportion of people, the disease may follow a cyclical waxing and waning course with earlier reactivation initially arrested by the host delaying disease presentation. In addition, it is clear that the subclinical phase of active disease prior to clinical presentation may be several months, as evidenced by the demonstration of culture positivity in asymptomatic persons. In HIV-infected persons in high burden settings, prevalent asymptomatic TB has been shown to be present in up to 8.5% [69].

### (b) Immunosuppression

A number of conditions are associated with increased risk of progression of TB, with HIV infection and anti-tumour necrosis factor (TNF) therapy being two well-documented examples. The effect of anti-TNF therapy is particularly striking in the macaque model of latent TB treatment, with anti-TNF resulting in almost universal reactivation in animals that had initially no signs or symptoms of active disease for at least six months from the time of infection [70]. In humans treated with anti-TNF therapy, especially with infliximab, the risk of TB is increased initially up to 20-fold with 43% of TB cases occurring within the first 90 days of administration of anti-TNF therapy, demonstrating how rapidly active disease can be precipitated [71]. However, reactivation is by no means universal. In an evaluation of the implementation of LTBI screening prior to anti-TNF therapy in Spain, 56 patients with positive TST (more than 5 mm) were identified who did not receive any isoniazid prophylaxis and in only one case did TB occur following anti-TNF treatment [72]. The impact of HIV on latent infection can be more challenging to evaluate as the majority of HIV/TB studies are performed in high burden settings where reinfection complicates understanding of the natural history of a single infectious episode. In studies in low burden settings, widespread ART use can also be a confounder. In addition and in contrast to anti-TNF therapy, HIV-associated immunosuppression is slowly progressive. In studies from the high burden setting of the South African mines, the risk of TB infection has been shown to double within the first year, with Mtb strains significantly more likely to be unique within 2 years of HIV-seroconversion than later on in the disease, suggesting that TB may more likely be precipitated by reactivation early in the course of HIV and that recent reinfection with subsequent rapid progression occurs in more advanced immunosuppression [73]. Studies from low prevalence settings (Spain and Switzerland) suggest that the rate of TB in HIV-infected persons who are TST positive but untreated reduces over time, with most of the excess cases of TB compared with TST negative occurring within the first 2 years of follow-up; in total, only 10–12% of this very high-risk group developed TB over a follow-up period of up to 5 years [74,75].

Box 2. **The nature of Mtb in latent infection.**

Mtb adapts to environmental triggers such as hypoxia, nutrient starvation and reduced pH encountered during infection by altering metabolism and arresting replication [49]. Adaptation often includes transient transcriptional activation of a characteristic set of approximately 50 genes under the control of the DosR ‘dormancy’ regulator, together with additional genes appropriate to the specific environmental cue [50]. The products of these induced genes are currently being explored as potential biomarkers. Resumption of replication following exposure to a more favourable environment is presumed to involve analogous transcriptional and metabolic reprogramming, including cell wall changes mediated by a family of transglycosylase enzymes [51]. The ability of Mtb to persist in a reversible non-replicating state is a key virulence factor but the direct equation of clinical latency with non-replicating mycobacteria and active disease with replicating mycobacteria is an oversimplification. Although active disease is characterized by uncontrolled increases in bacillary numbers, imaging and autopsy studies show that there are numerous micro-environments that exhibit varying degrees of progression and healing. A partially overlapping heterogeneous spectrum is seen in latent stages of infection. The prolonged courses of treatment required to prevent relapse following treatment of active TB are thought to be due to persistent populations of bacilli, whereas the efficacy of isoniazid as preventive therapy in latent infection is thought to be due to its effect on replicating bacilli. In short, although absolute numbers and proportion clearly differ it seems likely that both replicating and non-replicating bacilli are present in both latent infection and active disease.

### (c) Protection from reinfection

Although latently infected persons are at greater risk of developing disease through reactivation than are those uninfected there is some evidence that at least a proportion are protected against subsequent (re)infection. Andrews *et al*. [76] reviewed 18 studies in which 19 886 persons with or without latent TB, as evidenced by tuberculin reactivity, were followed up for active disease in the absence of intervention. The majority of these studies were published before 1950, and largely involved nursing and medical students entering clinical practice and exposed to extremely high annual rates of infection (median 33.6%). The incidence of disease in those with LTBI at entry was 5.1/1000 person-years and in those uninfected at entry was 18.2/1000 person-years. Once adjustments were made for reactivation and average timing of infection, risk reduction in those with LTBI was found to be 79%. Historical healthcare settings clearly represent an extreme scenario, however other approaches have also suggested a protective effect of infection. Using national datasets over long periods of time to model the dynamics of TB, Vynnycky & Fine [60] from data for England and Wales 1900–1990 predicted a 16–41% protection from an initial infection against reinfection, and Sutherland *et al*. [77] from data for the Netherlands predicted 63–81% protection. Taking an alternative strategy, Brooks-Pollock *et al*. [78] used cross-sectional household data from Lima, Peru from 1996–2002 to propose 35% protection. In detailed studies of reinfection in the rabbit model, Lurie [79] demonstrated that control of a re-infecting strain in previously infected rabbits was mediated by tissue resident mononuclear cells, with efficiency of control relating to the extent of the primary lesion from the initial infection. The mechanism of protection in humans is not known but clearly an improved understanding of this could greatly facilitate vaccine development.

## 5. Integrating the spectrum of tuberculosis with natural history

**Figure 3.**
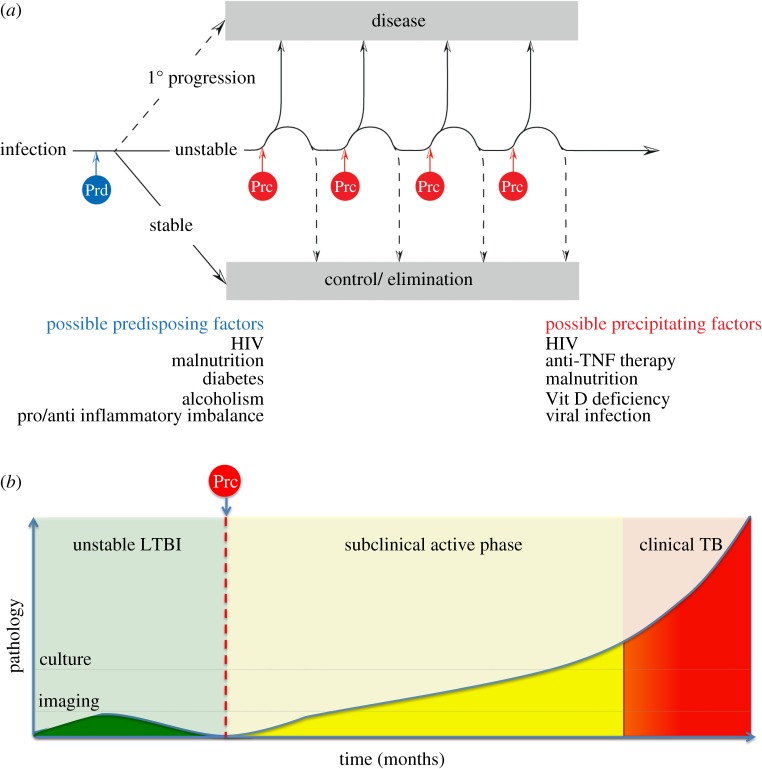
(*a*) Following infection, there may be a critical period where fate of infection is determined with predisposing factors (Prd) influencing this outcome. In a small proportion, the primary infection may be progressive; in those that control primary infection, a proportion may eliminate TB or exert highly effective control and be at very low risk of reactivation. In the third group, control may be unstable waxing and waning in response to a variety of precipitating factors (Prc) with reactivation of TB most likely to occur in this high-risk group. (*b*) Precipitating factors (Prc) may lead to progression of disease. Prior to presentation these individuals may pass through a subclinical phase of active infection which may last months; during this phase Mtb may be isolated by culture or pathology may be visible through imaging prior to symptomatic presentation. (Online version in colour.)

We and others have suggested that asymptomatic people considered to have latent TB might be better considered as part of a spectrum of infection states where at one end infection may have been eliminated, while at the other end disease may be active but in a subclinical form, and between these two extremes infection is controlled in a quiescent state [80–84]. When carefully considering the natural history of infection, it seems plausible that soon after initial infection and immune sensitization there are three main possible outcomes influenced by predisposing factors that determine the course of infection during this critical phase and alter the proportions in each group (figure 3). Some may initially develop primary progressive disease; this may be a very small proportion in adults but would likely be more common in advanced immunosuppression and infants. A second group (a high-risk group—the main group from which reactivation disease arises) enter a more unstable state with infection taking a waxing–waning course during which periods of progression triggered by precipitating factors may be followed by control (which may lead to evidence of immunopathology) or the development of clinical disease. Some precipitating factors may be more potent than others; very potent precipitating factors (such as anti-TNF and HIV) may have the effect of causing rapid progression over a short time interval. It is also in this group that isoniazid preventive therapy may be most effective. A third group may rapidly and effectively control infection and eventually may even sterilize the organism and may be at extremely low risk of progressing to active disease even in the presence of precipitating factors.

### (a) Predisposing and precipitating factors

Some predisposing and precipitating factors may overlap (figure 3). Many predisposing factors from the host side are known and can be considered as generally immunosuppressive (HIV, malnutrition, chronic kidney disease, type 2 diabetes mellitus, etc.) but many more may be unknown, poorly characterized, have more subtle effects and may or may not be genetically mediated. In particular, rather than just immunosuppression alone it is becoming more apparent that the extremes of the immune response may lead to detrimental outcome in TB, with a more balanced response being optimal (the so-called ‘Goldilocks effect’); weak responses may lead to unopposed bacillary replication whereas aggressive responses may lead to tissue damage and necrosis which may provide a more favourable environment for the bacillus. One of the implications of this, when considering biomarker discovery for novel diagnostics (see below), is that there may be at least two distinct correlates of risk.

Leukotriene (LT) A_4_ hydrolase (LTA4H) mediates the balance of pro-inflammatory eicosanoid LTB_4_ and anti-inflammatory lipoxin A. Zebrafish larvae in which LTA4H is over- or under-expressed are made hyper-susceptible to *Mycobacterium marinum* infection compared with wild-type either by low levels of LTA4H resulting in increased lipoxin A and impaired TNFα production, or excessive LTA4H resulting in increased LTB_4_ and increased TNFα production [85]. Humans who are heterozygous for a single-nucleotide polymorphism of LTA4H promoter rs17525495 (C/T) appear to have the best clinical outcome from TB meningitis; those who are homozygotes for the T allele (T/T) have increased LTA4H expression and inflammatory cerebrospinal fluid, but derive significantly greater benefit from dexamethasone therapy compared with the C/C genotype [85–87]. In humans, plasma prostaglandin E2 and lipoxin A levels likewise correlate with disease susceptibility in a similar bimodal fashion with both insufficient and abundant responses tied to disease exacerbation (Mayer-Barber & Sher 2014, unpublished results, personal communication).

Another intriguing and poorly understood predisposing factor is age. It is a consistent and striking feature of TB that the age of infection affects the risk of subsequently developing disease [56,88]. Infants and young children, especially those less than 2 years, are at considerable risk of developing disease following infection. Older children (5–10 years old) have consistently been shown to be at the lowest risk of TB following infection especially, whereas peri-pubescent adolescents and young adults are at much greater risk of developing cavitary TB compared with children less than 10 years old [56,88]. It has been suggested that this may relate to the immunoendocrine effects mediated by the balance between dehydroepiandrosterone (DHEA—a precursor of sex steroids) and glucocorticoids [89]. DHEA levels start increasing from 7 years old, peak in early adulthood and reduce in older adults [88]. One of DHEA's many effects is as a glucocorticoid antagonist, and the cortisol : DHEA ratio has important immunological consequences. Recently, DHEA has been shown to influence dendritic cell function to promote Th1 responses by increasing interleukin (IL)-12 and diminishing IL-10 production following Mtb stimulation, with increased expression of MHCI, MHCII and CD86 expression resulting in enhanced T cell proliferation and IFNγ production [90]. So it seems plausible that pro-inflammatory responses in healthy adolescents and young adults have detrimental effects leading to cavitary disease, anti-inflammatory responses in infants lead to their inability to control replication and there is a more optimal balanced response in older children.

A further incompletely understood historical observation recently being revisited is the effect of monocyte : lymphocyte ratio (M : L) on risk of disease. It has been found in both animal models and clinical observation that extremes of both low and high M : L result in a greater risk of developing TB, but what is still not clear is whether this is a predisposing factor [91] or a marker of progressive disease as TB treatment normalizes the M : L ratio [92].

While some precipitating factors may be well known and potent resulting in rapid progression of the at risk group (e.g. anti-TNF therapy), some may just contribute to a fluctuating course triggering disease in a minority. To consider an example, an interesting observation is the seasonality of TB with increased case notification that can be 20–25% higher in spring/summer compared with autumn/winter [93–96]. This is striking for an infectious disease with relatively prolonged and variable incubation. In common with other respiratory pathogens, one explanation would be behavioural, with winter crowding leading to greater transmission, but modelling evidence and analysis of unique and clustered Mtb strains suggest that this cannot fully explain seasonality of TB [97,98]. A seasonal precipitating factor such as vitamin D deficiency or viral respiratory infection (e.g influenza) is an alternative explanation. Vitamin D, synthesized within the skin requiring UV light, acts as an immunomodulatory and anti-inflammatory agent primarily exerting its effect on the macrophage, facilitating enhanced control of mycobacteria through pleiotropic mechanisms [92,99]. A number of clinical observations provide some support for the role of vitamin D deficiency in inducing reactivation. TB patients are well documented to have significantly lower vitamin D levels than healthy household controls [100,101], and the spring/summer peak in TB notifications is preceded by a winter trough in vitamin D levels in Cape Town [102]. There is some evidence to suggest that seasonality is more pronounced in foreign-born cases (who may be at greater risk of vitamin D deficiency owing to skin tone) compared with native cases in Europe [103,104]. An alternative seasonal precipitant could be viral infection. It has recently been shown that the type 2 interferon (IFNγ) response critical for mycobacterial control can be impaired by the downstream effects of type 1 interferons (IFNα/β) [105]. It has therefore been hypothesized that viral respiratory infections inducing a type 1 interferon response could lead to reactivation by impairment of type 2 interferon facilitated control of Mtb. In the mouse model, mycobacterial growth is enhanced and survival decreased in mice previously exposed to influenza by a mechanism dependent on type 1 interferon signalling [106]. In addition, historical observations and modelling of the 1918 influenza pandemic suggest a negative impact of influenza on TB [107]. It is also possible that aside from host factors, variability of the bacillus may influence rate of progression and disease outcome, and it is worth noting that there is clear evidence for diversifying selection in genes of the bacillus whose functional roles are less than clear [108].

Having a fuller understanding of these predisposing and precipitating factors and the magnitude of their effects might allow us to consider the impact of novel intervention strategies: for instance, whether widespread or targeted vitamin D replacement and/or influenza vaccination may impact the incidence of TB.

## 6. Novel diagnostic and preventive treatment strategies for LTBI

**Figure 4.**
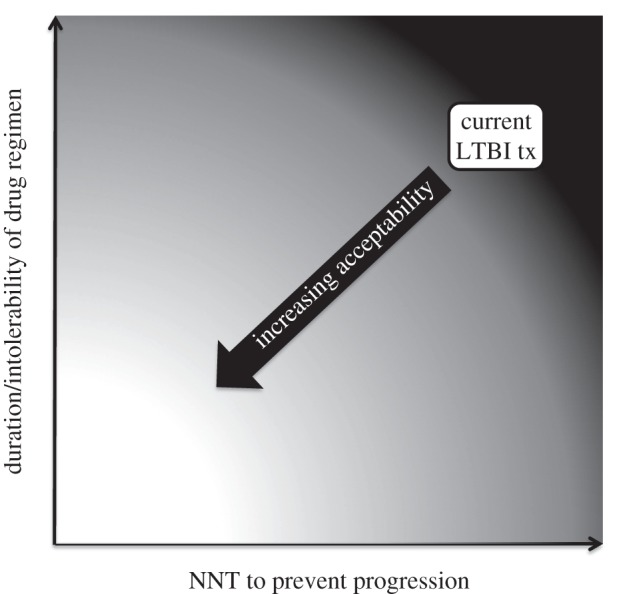
Acceptability of treatment relates to the duration and tolerability of treatment and the likelihood of benefit (prevention of progression to active disease). Improvements in drug regimens and/or improvements in predictability of diagnostic tests should lead to improved acceptability of treating LTBI.

Current approaches to management of latent TB centre on those at greatest epidemiological risk of progressing to TB, namely close contacts of active TB, HIV-infected and other persons about to undergo, or with established, immunosuppression. Following diagnosis of latent TB, preventive treatment is most commonly six to nine months of isoniazid or a three month course of rifampicin or rifapentine and isoniazid [82] (in HIV-uninfected persons). However, in many low- and middle-income countries with the highest burdens of TB, many of these high-risk groups do not receive preventive therapy, especially household contacts. In these settings, operational research may enhance the better implementation of clinical trials evidence and recommendations. However, to make significant progress towards TB elimination will also require providing preventive treatment to an even wider group of latently infected persons. One of the critical barriers to this is the acceptability of the current intervention to individuals at risk as well as healthcare professionals and policy makers. This is to a large extent influenced by the prolonged duration of treatment and the high NNT to prevent a case of active disease; hence acceptability should improve if either or both of these are improved (figure 4). An inexpensive on-the-spot diagnostic that provided an accurate assessment of likelihood of progression coupled with a single-dose prophylactic would be game-changing for TB control and enable mass testing and treatment programmes with a realistic chance of achieving eradication.

More predictive diagnostic tests are a goal of TB research agendas but how these tests might be implemented in practice also requires careful evaluation. The perfect diagnostic for latent TB would be a cheap, low resource, point-of-care test with very high positive and negative predictive value that maintained sensitivity in immunocompromised persons, notably HIV infection and children. However, it is worth noting that a single test cannot be both highly predictive for active TB and a sensitive marker of exposure as these test characteristics are to a degree a mutually exclusive. Another consideration is over what period of time should these tests be predictive? It may be substantially easier to develop tests (from both a development and validation perspective) that are predictive over a short period of time—e.g. risk over 12 months rather than predictive of life time risk—as it may be over this time frame that transition into a subclinical phase prior to symptomatic presentation occurs with characteristic changes in the host response and bacillary numbers and metabolic state. Such shorter term predictive tests may be of particular use in high burden settings where reinfection is common and in HIV-infected persons and other immunocompromised groups (such as in type 2 diabetes mellitus and chronic renal failure) where regular contact with healthcare many allow for regular screening. In addition, recent contacts of TB could be followed up annually during the period of greatest risk. Optimal test characteristics might change as the elimination phase progresses; understanding these requirements will be helped greatly by models showing the impact of different types of tests in different settings.

### (a) Markers of exposure

As discussed, TST and IGRA are poorly predictive but also are suboptimal measures of Mtb exposure. Both TST and IGRA show evidence of spontaneous reversion over time and hence some people previously exposed to and immune sensitized by Mtb may have a negative test. A proportion of these individuals may be identified using a two-step testing strategy where the initial negative TST boosts the immune response so subsequent TST or IGRA reverts to positive [109]. In addition, the sensitivity of TST and IGRA for active TB infection is only 70–90% [110–112]; not withstanding arguments as to why immunodiagnostic tests might be compromised in someone with active TB, a test that is not able to identify the most heavily infected individuals leaves room for improvement. A more sensitive test for Mtb exposure would give a better understanding of TB transmission dynamics and more accurate estimation of the annual rate of infection. Because IGRAs detect IFN-γ production after short-term incubation (16–24 h), they identify primarily ESAT6/CFP10-specific effector cells and it is possible that prolonged assays measuring alternative cytokines or flow cytometric assays could identify central memory populations that may be a better marker of exposure and history of immune sensitization than the currently available tests.

### (b) Predictive markers

Predictive biomarkers could be developed using two broad approaches. Careful follow-up of an at-risk population over time during which some develop TB might allow identification of biomarkers that were correlates of risk, although such natural history approaches would not only require very large numbers but would also have considerable ethical considerations unless carried out in groups where observation is standard of care, such as contacts of multi-drug resistant (MDR)-TB, where guidelines do not recommend preventive therapy (e.g. over 35-year-olds in some countries) or in placebo arms of intervention studies. Another approach would be to identify biomarkers for people at the transition of latent to active disease with subclinical and minimally active pathology or evidence of immunopathology (e.g. fibrotic scaring) and then validate these markers prospectively. The most useful predictive biomarkers will most likely be mycobacterial products or markers of host response identified within blood or urine or through skin testing. ‘Omics’ approaches are useful as exploratory tools to identify key components of diagnostic tests; transcriptomic approaches have been particularly successful at differentiating the extremes of active and latent TB [113,114], but these signatures may not be predictive of active TB if the signatures relate to response to disease process itself. More predictive tests would certainly be a huge advance allowing treatment decisions to be based on a biological as well as epidemiological markers of risk, but it is sobering to note how slow progress in the cancer field has been in developing markers of early detection into diagnostic tests. Such biomarkers will be challenging to develop, requiring a large amount of support to move down the pipeline from concept through to development, validation and implementation; but unlike vaccine, drug and diagnostic tests for active TB, no such pipeline exists for predictive tests for latent TB.

### (c) Drugs

Shorter drug regimens are highly desirable for treatment of both latent and active TB, with the current prolonged treatment duration required to ensure that recrudescence of persisting organisms does not occur after cessation of therapy. In addition, a prolonged treatment course is associated with poor treatment adherence in routine settings. Drugs that contribute most to treatment shortening of active or latent TB (rifampicin and pyrazinamide) have the most potent sterilizing ability (usually determined by evaluating relapse rate in the murine model), whereas isoniazid, although rapidly bactericidal, largely acts on replicating bacilli and has poor sterilizing activity [115]. As an alternative strategy to shortening treatment for LTBI, rather than targeting persisting organisms, might be to provoke resuscitation of non-replicating bacilli and couple this with rapidly bactericidal therapies such as isoniazid. Such approaches will require a far more sophisticated understanding of the mechanisms of resuscitation and ability to define the metabolic states of single organisms.

The optimal duration of isoniazid as preventive therapy is nine months [116]; with addition of rifampicin or rifapentine this reduces to three months and rifampicin combined with pyrazinamide is effective after two months of administration (although unacceptable toxicity prevents widespread use of this regimen) [117]. There are several novel or re-purposed drugs in later stages of the TB drug pipeline [118] that have impressive sterilizing ability either alone or in drug combinations (usually in pyrazinamide-containing regimens). Bedaqualine (recently FDA approved for active MDR-TB) and sutezolid (an oxalozidinone in phase 2 studies) seem most promising in this respect. Nitroimidazole derivatives (delamanid and PA-824) and moxifloxacin are also possibilities [119]. Whether reductions in treatment duration beyond two months could be feasible is not certain, however it is possible that this pipeline provides several options for preventive treatment in drug-resistant TB contacts. A major consideration for novel regimens in addition to cost [120] is toxicity and side effects, which are a major factor when treating otherwise asymptomatic persons (as demonstrated by pyrazinamide), especially while we are unable to more precisely define who will derive greatest benefit from preventive therapy.

A further challenge is how to best evaluate novel LTBI regimens. Currently, the only endpoint for clinical trials is the absence of disease and hence in order to demonstrate clinical efficacy studies they will need large numbers and prolonged follow-up. A surrogate marker of clinical response (analogous to 14-day early bactericidal activity or two-month culture conversion for active TB) may allow for more rapid evaluation of different regimens to select which should go forward to larger clinical studies. Peripheral blood biomarkers that signify treatment success for LTBI would be very useful in this regard. An alternative approach that is being developed similar to oncology studies is the use of PET/CT imaging to evaluate response of therapy. 18F-Fluorodeoxyglucose (FDG) is the most widely used tracer, is a non-specific marker of metabolic activity and is taken up avidly by activated neutrophils and macrophages [121]. Sites of active TB even in the absence of symptoms accumulate FDG avidly and a number of studies have demonstrated that uptake is markedly reduced following TB treatment [122–125]. Tracers that are more specific for Mtb would be a great advance and these are currently in early stages of development.

### (d) Post-exposure vaccines and immunomodulation

TB vaccines can be administered either pre-infection, designed to prevent infection from occurring, or post-infection, designed to prevent latent infection progressing. H56 is a multistage vaccine comprising the Ag85B, ESAT-6 and Rv2660 antigens, is one of the first vaccines to be designed to be used post-infection, and is soon entering phase I/IIa studies in South Africa in latently infected and uninfected adults [126].

However, recently the MVA85A vaccine, a novel TB vaccine (administered pre-infection) that was in the most advanced stage of clinical development, yielded disappointing results that demonstrated safety but no significant efficacy over placebo in preventing TB when administered to BCG-vaccinated, HIV-negative infants with no evidence of latent TB infection, in a high burden setting [127]. The resulting debate has highlighted how limited our understanding of protective immunity in humans is and the dangers of extrapolating findings in animal models to clinical practice [128]. Our understanding of how to use a vaccine to safely prevent reactivation of an established latent infection is even more limited, as there are fewer appropriate animal models and there is always concern about the induction of Koch reactions, where rapid induction of vigorous immune response in persons with an asymptomatic subclinical infection may result in immunopathology and symptomatic deterioration. It is critical that we develop a better understanding of what the immunological correlates of protection from disease are in humans to inform vaccine design. One approach may be in the careful evaluation of the heterogeneous effects of vaccination either within a single trial or between trials performed in different populations and the correlation with clinical outcomes. Another approach would be to try and better understand the protective effects of natural infection especially the immunological basis for this (see above), or to characterize the immunological differences in response to infection between older children and young adults, who have the best and worse outcomes following infection, respectively (see above). In particular, if we are to develop vaccines that prevent reactivation or successfully eliminate latent infection, we need to better understand the immunological mechanisms that precipitate reactivation and control in those with unstable latent infection, which may require refinement of existing animal models to more accurately reflect the natural history of TB in humans.

An alternative and innovative approach would be to combine immunomodulation with anti-tuberculous treatment as a method to shorten therapy. This immunomodulation could take the form of either a vaccine or a drug. RUTI is a novel vaccine comprising heat inactivated, liposomed fragments of Mtb grown under different conditions of stress designed to be administered after one month of chemotherapy of LTBI and facilitate immune clearance of persisting bacilli [129]. The vaccine is currently in phase 2 studies in HIV-infected and -uninfected persons with LTBI (http://clinicaltrials.gov/show/NCT01136161)

## 7. Concluding remarks

A coordinated strategy will be required to effectively tackle the reservoir of latent infection. Improved data are needed to more accurately estimate the scale of the problem and quantify the number of new infections occurring each year, and a redoubling of effort will be required to reduce this as far as possible by implementing currently recommended interventions. However, in order for widespread treatment of latent TB to be acceptable to the public, healthcare providers and policy makers, major advances on the currently available diagnostic and interventional tools will be required. Progress in identifying who is most likely to reactivate and how this occurs will assist the development of more predictive diagnostic tests allowing interventions to be focused on those that will benefit most. The development of drugs that effectively target and rapidly sterilize the subset of persistent bacilli should allow for significant reductions in the duration of preventive treatment. Both of these should improve acceptability of more widespread treatment of latent infection. In addition, greater understanding of who is protected from reinfection and how this occurs would provide key pieces of knowledge to facilitate progress with development of effective vaccines and immunomodulatory agents that could have a major impact.

The aim to eliminate TB by 2050 is a bold one and the development of the post-2015 TB strategy and targets provides an opportunity to identify the critical gaps in our knowledge and to focus the scientific community, policy makers, advocates and funding agencies on achieving this challenging goal.
